# Surgical outcomes in cardiogenic shock patients with preoperative extracorporeal membrane oxygenation (ECMO)

**DOI:** 10.1186/s13019-021-01542-7

**Published:** 2021-08-03

**Authors:** Mahmoud Yousef Ibrahim Abuharb, Dong Ran, Zheng Jubing, Liu Taoshuai, Dong Haiming, Hou Xiaotong, Song Yue, Zhao Yang, Li Yang

**Affiliations:** Cardiovascular Surgery Department, Capital Medical University Affiliated Anzhen Hospital, Aiguo Road, Lianhe Avenue, Beijing, 100029 China

**Keywords:** Extracorporeal membrane oxygenation, Cardiogenic shock, Surgical treatment

## Abstract

**Objectives:**

To summarise the surgical outcomes in patients with cardiogenic shock supported by preoperative extracorporeal membrane oxygenation (ECMO).

**Methods:**

Between May 2012 and August 2017, eight patients with cardiogenic shock, who were supported by ECMO, underwent emergency surgery; four of them had isolated coronary artery bypass grafting, three had coronary artery bypass grafting with mitral replacement, and one had mitral valve replacement with left ventricular posterior wall repair.

**Results:**

All eight patients were successfully weaned off from ECMO after their surgeries. Postoperative ECMO time ranged from 6.8 to 228.0 h, with a median of 68.4 h. Two patients died postoperatively while another six survived. The follow up time for the six patients ranged from three to 66 months, whereby one of them died in the third month due to septicaemia. The remaining five patients survived with good cardiac function based on the NYHA classification.

**Conclusion:**

ECMO is a vital bridge in the preparation of critically-ill patients for cardiac surgery. It is associated with acceptable outcomes among most of the patients.

## Introduction

Extracorporeal Membrane Oxygenation (ECMO) was first applied clinically in 1973 [1, 2]. Currently, ECMO is mainly used as circulatory assistance to rescue patients with acute circulatory and/or respiratory failure. Its two main purposes are to serve as a bridge-to-recovery (BTR) in patients who are suffering from heart failure and secondly, as a bridge-to-next decision (BTD) where it serves as a temporary intervention until further clinical decisions can be made. The examples of BTD include surgery, interventional treatment, long-term ventricular assist device (LVAD) implantation, heart transplantation [3], etc. To date, there are limited published studies on the surgical outcomes of cardiogenic shock patients supported by ECMO. We aimed to contribute to the literature by reporting on the ECMO cases in the Cardiac Surgery Department of Beijing Anzhen Hospital and to evaluate the surgical efficacy of ECMO on cardiac patients.

## Materials and methods

This study was conducted from May 2012 to August 2017. All the patients with cardiogenic shock who received ECMO before emergency surgery were included (total of 8 patients). Patients with the following clinical signs would be considered for ECMO support: systolic blood pressure < 90 mmHg, required two or more high-dose vasopressors, intra-aortic balloon pump counterpulsation (IABP) needed to maintain blood pressure at 90 mmHg, peripheral vascular constriction, cold extremities, irritability, altered mental state, urine output < 30 ml/h. High-dose vasopressor included dopamine ≥10 μg/kg/min, adrenaline ≥0.05 μg/kg/min, dobutamine ≥1 μg/kg/min, or milrinone ≥0.5 μg/kg/min.

A total of eight patients were included. Preoperatively, all eight patients were put on ECMO using Maquet (2050 package) or Medtronic pump head, as well as the Medos membrane lung combination. The MO loop was prefilled with lactated Ringer’s solution or normal saline. The pulse-arterial ECMO (VA-ECMO) mode provided circulatory assistance through the femoral arteriovenous catheters. The intubation were all made of Medtronic Carmeda coating (19 Fr or 21 Fr for vein, 15 Fr or 17 Fr for artery). Among these patients, two received percutaneous puncture catheterisation and six underwent incision catheterisation with the placement of distal irrigation. The ECMO operation was performed in the catheterisation lab for three of the patients, and in the EICU for another four patients. One patient underwent the procedure in the normal ward.

During ECMO, close monitoring was conducted every four hours to maintain the activated coagulation time (ACT) at 180–220 s and the activated partial thromboplastin time (APTT) at 50–60 s. Appropriate sedatives such as fentanyl, propofol, and dexmedetomidine were added to the intravenous pumps. The patients’ conditions were closely monitored for any signs of recovery. All of the eight patients were supported with ECMO and mechanical ventilation. A protective lung ventilation strategy was adopted and the ventilator parameters were adjusted based on the blood gas results. When the patients showed good lung functions, sedation would be stopped and patients would be extubated. However, one of them awakened and had to proceed with ECMO without sedation. Bedside echocardiography was performed daily to assess the patients’ cardiac function. If patients became haemodynamically stable, ECMO would be stopped for one hour. If the patients continued to be stable with good blood circulation and cardiac function, ECMO would be removed.

Operatively, all the eight patients underwent median sternotomy under general anaesthesia. Four patients underwent off-pump coronary artery bypass grafting (CABG) while on ECMO with an intraoperative ACT of > 380 s. The other four patients underwent on-pump CABG coronary artery bypass grafting and they were heparinised to achieve ACT > 480 s. Conventional ascending aorta cannulation, as well as superior and inferior vena cava cannulation, were established. The ECMO cannula was disconnected from the loop. Following that, the loop would be attached to a connector to turn it into a closed-loop self-circulation. The aortic cross-clamp was applied before starting the cardioplegia solution by cooling the heart surface with ice debris.

Surgically, three patients underwent coronary artery bypass surgery and mitral valve replacement. Another patient underwent routine mitral valve replacement with left ventricular posterior wall repair. In the operation, a sidewall clamp was first applied on the aorta while the proximal stapler was used to anastomose the proximal end of the target vessel before being moved down to perform distal anastomosis. The other four patients underwent simple CABG. All the bridging vessels used were the great saphenous veins. Of the total 15 grafts, five were for the anterior descending branch, one was for the diagonal branch, six for the circumflex branches, and three for the posterior descending branches. Mitral valve replacement was performed in four patients in which the posterior leaflet was retained and the intermittent suture was implanted. An artificial mechanical valve was used (Medtronic AP360 M24 or 26) for the patient with a ruptured posterior wall of the left ventricle as a result of visceral trauma. The valve was repaired with the intracardiac interrupted suture combined with the epicardial “sandwich” suture. All the patients were followed-up on November 30th 2017, via phone and by outpatient visits to determine their survivals and cardiac functions.

## Results

There were six males and two females with an age range between 15 and 78 years-old (55.5 ± 15.5) and a weight range between 60 and 100 kg. The preoperative ejection fraction was between 28 and 70% (52.0 ± 14.0). The left ventricular end-diastolic diameter ranged between 43 and 65 (54 ± 7) mm and the left ventricular end-systolic diameter was between 43 and 65 (35 ± 7) mm. Among them, six patients had an acute myocardial infarction, one patient had unstable angina, while another suffered from cardiac trauma. Three of them received cardiopulmonary resuscitation (Table 1).

**Table 1:** Preoperative characteristics of the patients

Patient	1	2	3	4	5	6	7	8
Gender	male	male	male	male	female	female	male	male
Age	59	63	78	15	62	56	47	64
Height (cm)	175	175	170	170	175	160	175	168
Weight (kg)	90	85	70	60	73	60	100	60
Diagnosis	AMI	AMI	unstable angina	cardiac trauma (posterior left ventricular rupture)	AMI	AMI	AMI	AMI
MR	none	none	moderate	severe	severe	none	severe	severe
CPR history	none	yes	yes	none	none	yes	none	none
DM	none	none	yes	none	none	one	none	none
HTN	yes	yes	yes	none	none	yes	yes	none
Hyperlipidaemia	yes	yes	none	none	none	yes	none	none
EF(%)	43	28	54	60	70	65	52	43
LVDD	50	49	50	60	55	43	65	59

*AMI* Acute Myocardial Infarction, *MR* Mitral Regurgitation, *EF* Ejection Fraction, *LVDD* Left Ventricular End Diastolic Diameter

*Mitral Regurgitation was due to papillary muscle rupture

**Table:** 

n	8
Male	6
Age Range	47–78
Resuscitation	4
Diabetes	1
Hypertension	6
Moderate-Severe Mitral Regurgitation	5
EF	52 ± 13
LVDD	53 ± 6

The preoperative ECMO time ranged from 0.5 to 72.5 (24.2 ± 29.8) hours. The mean surgical time was 254.4 ± 56.6 min whereas the extracorporeal circulation time and the aortic block time were slightly lower at 139.3 ± 34.0 min and 92.5 ± 24.1 min respectively. Perioperative blood investigation showed red blood cells 6–46 U (median 11.5 U), plasma 400–2800 ml (median 900 ml), platelets 0–10 U (median 2.5 U). Lastly, the postoperative-ventilator assistance time ranged from 37 to 546 h, with a median of 162 h.

All eight patients were successfully weaned off ECMO and ventilation. However, only six patients survived and were discharged from hospital, thus giving a survival rate of 75%. Postoperative ECMO assistance time ranged from 6.8 to 228 h with a median of 68.4 h. There were two deaths (25.0%) during the perioperative period, one of them died of septicaemia and multiple organ failure while another died of a stroke and multiple organ function. In addition, five patients (62.5%) experienced renal failure after the operation and required dialysis. Two patients suffered from lung infections (25.0%) and upper gastrointestinal bleeding (25.0%) respectively. Type I respiratory failure occurred in one patient (12.5%) and acute liver failure also occurred in one patient (12.5%). All six cases were followed up after the operation. The follow up time ranged from 3 to 66 months with a mean of 12.5 months. However, one patient died of septicaemia three and a half months after the operation. The remaining five patients showed good cardiac functions with grade I NYHA classification (Table 2).

**Table 2:** Postoperative clinical characteristics of the patients

Patient	1	2	3	4	5	6	7	8
Pre-operative ECMO time (h)	72.5	29.6	0.5	66.7	18	3.7	2	0.7
Post-operative ECMO time (h)	16.5	136.4	66	6.8	135.5	70.8	228	40.3
ECMO total time (h)	89	166	66.5	73.5	117.5	74.5	230	41
Type of surgery	CABG	CABG	CABG	Left ventricular posterior wall repair + MVR	CABG +MVR	CABG	CABG + MVR	CABG + MVR
Pre-Operative IABP	Yes	None	Yes	None	None	None	None	None
Operation time (min)	236	270	195	275	255	170	345	305
CPB time (min)				121	118		190	128
Cross-clamp time (min)				87	78		128	77
RBC (u)	6	46	11	8	12	16	46	8
Plasma (ml)	400	2800	400	1200	800	1000	2600	400
Platelet (u)	3	10	1	0	4	2	3	0
Mechanical ventilation time (h)	37	336	133	546	167	157	31	104
In-hospital death	None	Yes	None	None	None	None	Yes	None
Post-operative follow up time (month)	66	NA	17	17	8	5	NA	3
Follow up	Alive	Died in hospital	Died 3.5 months	Alive	Alive	Alive	Died in hospital	Alive
Follow up cardiac function (NYHA class)	Class 1	NA	NA	Class 1	Class 1	Class 1	NA	Class 1

*CPB* Cardiopulmonary Bypass, *CABG* Coronary artery bypass graft, *MVR* Mitral valve replacement, *h* Hour, *min* Minute, *IABP* Intra-aortic balloon pump, *ECMO* Extracorporeal membrane oxygenation, *NA* Not applicable

## Discussion

A significant reduction in cardiac output can cause severe acute circulatory failure that is associated with a high mortality rate. The rate of hospitalised death due to cardiogenic shock can be as high as 80%. Thus, it is important to reverse cardiogenic shock. In this study, emergency ECMO was used to assist the surgical treatment of cardiogenic shock. The subsequent in-hospital mortality rate was only 25.0%. Although ECMO is generally used for cardiogenic shock after cardiac surgery [4], a recent study by Bastan et al. [5] recruited 517 patients who received ECMO as a result of postoperative cardiogenic shock. The ECMO removal rate was 63% and the survival rate was 24.8%. The 6- and 12-month follow up survival rates were 17.6 and 16.5% respectively while the 5-year survival rate was 13.7%. According to the study, the risk factors for in-hospital death included old age, preoperative renal insufficiency, diabetes, obesity, intraoperative lactic acidosis, and high scores of the European Heart Surgery Risk Assessment System (EuroScore).

In this study, the patients with cardiogenic shock received ECMO before surgery. The ECMO removal rate was 100% and the survival rate at discharge was 75%. Thus, in comparison, preoperative implantation of ECMO is likely to confer a better prognosis than ECMO implantation after cardiac surgery. In a 2009 study by Bautista-Hernandez et al. [6], the surgical mortality was 38% among 26 patients who received a preoperative application of ECMO for congenital heart disease. Some of the complications of ECMO included death, inability to remove ECMO, and long-term dependence on ECMO. Another study in Taiwan reported a 50% death rate among 16 cases of CABG patients who received ECMO reports [7]. Higher mortality was observed among those over 60 years old and those with severe hypoxic encephalopathy.

There are several limitations to this study. This was a retrospective study with a small number of cases.

## Conclusion

In short, preoperative ECMO support in patients with cardiogenic shock provide critically-ill patients with opportunities for surgical treatment. It is also associated with better surgical outcomes.

## Data Availability

All data are available upon request from the Department of Cardiothoracic Surgery at Beijing Anzhen hospital.

## Abbreviations

ECMO: Extracorporeal Membrane Oxygenation
NYHA: New York Heart Association
BTR: Bridge-to-Recovery
BTD: Bridge-to-Next Decision
ACT: Activated Coagulation Time
APTT: Activated Partial Thromboplastin Time
IABP: Intra-aortic Balloon Pump Counterpulsation
VA-ECMO: Veno-Arterial Extracorporeal Membrane Oxygenation
EICU: Electronic Intensive Care Unit
Cm: Centimetre
Kg: Kilogram
MR: Mitral Regurgitation
CPR: Cardiopulmonary Resuscitation
DM: Diabetes Mellitus
HTN: Hypertension
EF: Ejection fraction
LVDD: Left Ventricular End Diastolic Diameter
AMI: Acute Myocardial Infarction
N: Number
Min: Minute
H: Hour
Ml: Millilitre
U: Unit
CPB: Cardiopulmonary Bypass
MVR: Mitral Valve Replacement
EuroScore: European Heart Surgery Risk Assessment System
